# Mutational Analysis of *EXT1*in a Chinese Family Affected by Hereditary Multiple Osteochondroma

**DOI:** 10.1155/2021/8888948

**Published:** 2021-08-06

**Authors:** Guangzhi Yuan, Qiang Su, Wenjun Liao, Wei Hou, Linke Huang, Peng Wang, Huayu Wu

**Affiliations:** ^1^Orthopedics Department, The Second Affiliated Hospital of Guangxi Medical University, Nanning, Guangxi, China; ^2^Guangxi Institute for Food and Drug Control, Guangxi, China; ^3^Department of Cell Biology and Genetics, Guangxi Medical University, Nanning, Guangxi, China; ^4^Department of Physical Examination/Health Management Department, First Affiliated Hospital of Guangxi Medical University, Nanning, Guangxi, China; ^5^Key Laboratory of Ministry of Education for Gastrointestinal Cancer, School of Basic Medical Sciences, Fujian Medical University, Fuzhou, Fujian, China

## Abstract

**Objectives:**

To discuss the mutational features and their relationships with disease in a family with hereditary multiple osteochondroma (HMO) from Guangxi Province (GXBB-1 family), China.

**Methods:**

Genomic DNA and total mRNA were extracted from peripheral blood cells of GXBB-1 family members. Whole elements of the *EXT1*gene and its transcript, including exons, introns, exon-intron boundaries, and coding sequence (CDS) clones, were amplified and sequenced. Allele-specific PCR was used to confirm the position and type of mutation.

**Results:**

All patients from the GXBB-1 family harbored the cosegregating heterozygous c.1056+1G>A mutation located in *EXT1*at an exon-intron boundary. Another three single-nucleotide polymorphisms (SNPs) were also detected in the patients, including IVS2+1G>A in intron 2, c.1844 T>C [p.Pro (CCT) 614Pro (CCC)] in exon 3, and c.2534G>A [p.Glu (GAG) 844Glu (GAA)] in exon 9. The latter two SNPs were synonymous variations.

**Conclusions:**

The heterozygous c.1056+1G>A mutation cosegregated with the phenotype, indicating that it is a pathogenic mutation in the GXBB-1 family. This mutation is reported for the first time in Chinese HMO patients. IVS2+1G>A and c.2534G>A have no relationship with the occurrence of disease. However, c.1844 T>C and c.1056+1G>A are linked, and their interaction needs to be further studied. c.1844T>C is a new SNP that has not been reported internationally.

## 1. Introduction

Hereditary multiple osteochondromas (HMOs), previously called hereditary multiple exostoses (HMEs), are rare autosomal dominant developmental bone disorders that usually affect cartilage ossification and are characterized by multiple benign osteocartilaginous masses that grow outward from the metaphyses of long bones (exostosis cartilaginea), such as the ends of femurs and tibias in the lower limbs or the humerus and forearm of the upper limbs [1]. HMO patients often have shortened stature, bony deformities, and restricted joint motion. Nearly all HMO patients harbor pathogenic mutations in the *EXT* gene family. Approximately 56-78% and 21-44% of cases are caused by genetic mutations in *EXT1* and *EXT2*, respectively [2]. However, the mutational frequencies in these two genes vary among ethnic groups. In the Western population, mutations in the *EXT1* and *EXT2* genes are responsible for 40%~75% and 20%–40% of HMO cases, respectively [3–5], compared with 14~53% and 33% ~40% in the Chinese population, respectively [6, 7].

Boyer described the first HMO patient in 1814. As of 2008, 157 and 71 mutations in *EXT1* and *EXT2* related to HMO had been reported, respectively. By 2011, the corresponding numbers had increased to 170 and 84, respectively. By 2013 and 2018, the numbers had increased to 429 and 223 and to 463 and 230, respectively [8–10].

We performed genetic analysis in a four-generation HMO family from Guangxi Province to clarify the clinical features, pathogenic mutations, and mutation types and to accumulate more information for the diagnosis and treatment of HMO.

## 2. Subjects and Methods

### 2.1. Subjects

**Figure 1:**
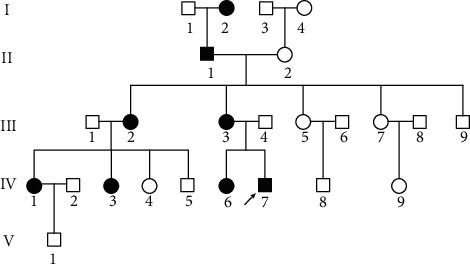
The Pedigree of the GXBB-1family with multiple hereditary osteochondromas from Guangxi Province. The proband (IV-7) is noted with a black arrow. ■: affected male; ●: affected female; □: healthy male; ○: healthy female.

The proband (IV-7) was a ten-year-old boy in a multiple generation HMO family from the eastern region of Guangxi Province, China (GXBB-1). The distal ulna and radius, distal femur, and proximal tibia were observed to have multiple exostoses, and pathological diagnosis supported osteochondroma in this boy. All the other members of the GXBB-1 family were also examined according to International Diagnostic Standards in Figure 1 [3].

### 2.2. Mutation Screening

Genomic DNA and RNA were extracted from peripheral blood samples according to the manufacturer's instructions. cDNA was obtained by reverse transcription using RNA as a template. The coding sequence (CDS) of EXT1 was amplified using the following two primer pairs: EXT1aH-1223F/EXT1aH-1223R (5′-AGCTGAAAGTGTTGATTGGGA-3′/5′-CTCATCGCCTATGACGGCAG-3′) and EXT1bH-1293F/EXT1bH-1293F (5′-TTGGGTCCTTCAGATTCCTGG-3′/5′-TGGATCTGCACTGGGAAGAGA-3′). The amplification consisted of the following protocol: predenaturation for 4 min at 95°C followed by 30 cycles of denaturation at 94°C for 40 s, annealing for 50 s at 58°C, and extension for 5 min at 72°C; and finally, extension at 72°C for 7 min. The PCR products were purified, cloned, and sequenced at the Beijing Genomics Institute (BGI), Shenzhen.

Based on the CDS results for the *EXT1* gene and the sequence of this gene (GenBank: NC_000008.11), the target gene fragment was amplified by PCR using EXT1-649F (GCAAAGACTGGGCAAACCAA) and EXT1-649R (AGGCCAAGCTGGCAATTAGAT). PCR programs included an initial denaturation of 5 min at 95°C, followed by 30 cycles of 40 s at 94°C, 50 s at 57.5°C, and 1 min at 72°C. The PCR products were sequenced at the Beijing Genomics Institute (BGI), Shenzhen. To further identify the mutation in the *EXT1* gene in the family, allele-specific PCR (AS-PCR) was performed using the EXT1-402F (5-′GGAAGCAAAGACTGGGCAAAC-3′) and EXT1-402R (5′-AAGGCTCCAGGGCCTCTTAT-3′) primers. PCR programs included an initial denaturation of 4 min at 95°C, followed by 30 cycles of 40 s at 94°C, 50 s at 66°C, and 55 s at 72°C. PCR products were separated on a 1.5% polyacrylamide gel. Bands with a size of 402 bp were considered to have *EXT1* mutations.

## 3. Results

### 3.1. Sanger Sequencing of the EXT1 Gene CDS Clones

All patients harbored a heterozygous deletion in the second exon of the *EXT1* gene (NM_000127.2:c.1736-1829del), with only 87% alignment with reference sequences from the database (GenBank: NC_000008.11). This provided evidence that the patients had exon 2 skipping. Unaffected family members and normal controls had no deletions, resulting in 99% matching with the reference sequence.

### 3.2. Sanger Sequencing of the EXT1 Gene

**Figure 2:**
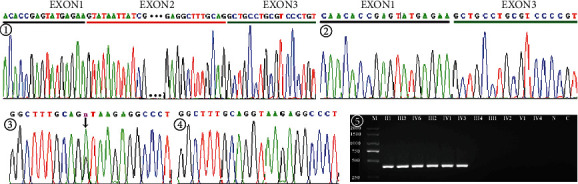
① III4 (unaffected), no exon deletion. ② IV6 (affected), exon 2 deletion. ③ IV6 c.1056+1G > T in *EXT1*. ④ III4, no frame-shift mutation. ⑤ Allele-specific PCR: affected (II1, III3, IV6, III2, IV1, IV3), unaffected (III4, III1, IV2, V1, IV4), and normal control (N, C).

As shown in Figure 2, all patients from the GXBB-1 family harbored a heterozygous splicing mutation (c.1056 G>A+1). The following three additional variations were also detected: (1) IVS2+1G>A in intron 2, (2) c.1844 T > C [p.Pro (CCT) 614Pro (CCC)] in exon 3, and (3) c.2534G > A [p.Glu (GAG) 844Glu (GAA)] in exon 9. The latter two SNPs were synonymous variations.

### 3.3. Allele-Specific PCR

The 3′ AS primer perfectly matched only the mutant allele. Therefore, a 402 bp fragment was amplified in the patients (II1, III3, IV6, III2, IV1, and IV3), while no products were detected in unaffected or normal controls.

### 3.4. Amino Acid Sequences of Wild-Type and Mutant Proteins

**Figure 3:**
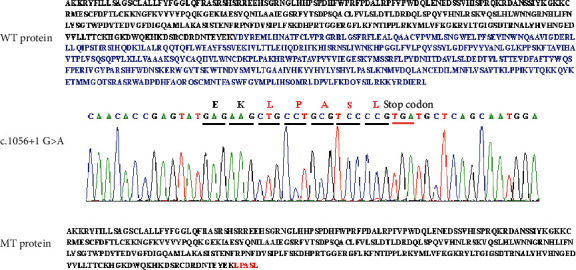
Amino acid sequences of wild-type and mutant proteins. The length of the wild protein is 746aa, while the mutant truncated protein is 326aa (p.Leu322TyrfsX6). The red sequences (LPASL) indicate new amino acids caused by the frame shift.

The original splicing site was abolished due to substitution of the first base G with base A in intron 2, which led to the exclusion of exon 2 in mature mRNA and premature termination at the protein level. We inferred that the truncated protein possessed 326 amino acids (p.Leu322TyrfsX6), with the last 5 amino acids being newly formed in Figure 3.

## 4. Discussion

HMO is highly genetically heterogeneous. The types and frequencies of mutations vary widely among ethnic groups, but the majority are missense mutations, nonsense mutations, splicing-change mutations, or small insertions or deletions [1]. Meanwhile, mutation hotspots occur, such as 1469delT in exon 6 and c.668G_C (p.R223P) in the *EXT2* gene [5, 11, 12] and p.Leu490Argfs∗9 and p.Arg340Leu in the *EXT1* gene [13, 14]. George A. suggested that the 11^th^ exon of *EXT1* was a mutation hotspot [6], and Zhuang reported three hotspot sequences prone to deletion residing in the 8^th^ exon. SCL found two novel mutation hotspots [5], namely, p.Ala409Profs∗26 and p.Ser290Ter and the highest frequency mutation p.Leu490Argfs∗9 in Brazilian HMO patients. The Brazilian population is a mixed population with a strong influence of Europeans in its colonization history, which could be responsible for the presence of recurrent alterations in hotspots in HMO patients from Brazil [15].

Nonsense, frameshift, and splicing-site mutations can directly or indirectly cause premature protein translation, finally leading to rapid protein degeneration or loss of function. However, missense mutations that change specific or several amino acids and impair protein stability or normal function affect the corresponding biosynthesis process and lead to abnormal chondrocyte proliferation.

In this report, the splicing mutation (c.1056+1G > A) was found to substitute the GA at the 5′ splicing donor site with AA, while the 3′ acceptor site remained unchanged. This led to removal of the entire 2^nd^ exon and intron after mRNA splicing, forming the truncated protein p.Leu322TyrfsX6. Compared with the wild-type protein, the mutant protein had fewer (420) amino acids. Both of the proteins shared the 1^st^~321^st^ amino acids, but the 322^nd^ to 326^th^ amino acids were different (Figure 3).

The *EXT1* protein contains four structural or functional domains, namely, signalP, transmembrane regions, exostosin (110~396 amino acids), and glycosyl transferase 64 (480~727 amino acids), which are very important for maintaining protein activities. The molecular mechanism underlying missense mutational effects on HMO phenotypes remains unclear, but the mechanisms for the other three types of mutations (nonsense, frameshift, and splicing-site mutations) seem obvious because all the mutations lead to partial or total removal of the exostosin and/or glycosyl transferase 64 protein domain [16, 17]. In our GXBB-1 family, the splicing-site substitution (c.1056+1G > A) resulted in partial and total removal of the exostosin and glycosyl transferase 64 domains, respectively. The coding product of *EXT1* and *EXT2*, i.e., heparan sulfate synthetase (HS), is a type II transmembrane protein crossing the surface of the endoplasmic reticulum. HS has glycosyltransferase activity and participates in the biosynthesis of heparan sulfate, which is crucial for normal skeletal development. *EXT1* and *EXT2* can form a protein heterodimer that has significantly higher biological activity than either of the proteins alone [18, 19]. The c.1056+1G > A mutation in the *EXT1* gene impairs the formation of an active heterodimer with *EXT2*, and this mutation cosegregated in the GXBB-1 family members.

In the past few years, many novel mutations in the *EXT* gene have been reported. In 2016, Daichi et al. found 22 novel mutations in the *EXT1* and *EXT2* genes in Japanese HMO families [20]. One year later, SCL et al. observed 31 novel mutations in Brazilian HMO families [16]. In China, Li et al. reported 12 novel mutations in *EXT1* and 14 novel mutations in *EXT2* among 73 HMO patients [8].

Deletions in *EXT1* are the most common mutation type, followed by many nonsense and missense mutations, but splicing mutations are relatively rare in Chinese patients. Only c.1417+1G > A, IVS5+1 G > A, IVS8+2 T > G, c.1883+1G > A, c.I8+2 T > G-8, c.1883+1G > A-9, and c.1883+2 T > A have been reported. The splicing mutation c.1056+1G > A was first reported in the Chinese population. A few families with the same mutations have also been reported/recorded in the United Kingdom, Belgium, and Brazil. Therefore, we do not consider this to be a common or hotspot mutation [4, 16, 21, 22].

The IVS2+1G > A variation in the GXBB-1 family is located in the *EXT1* intron rather than at a splicing site. The c.2534G > A variation has no apparent effects on osteochondroma. The IVS2+1G > A and c.2534G > A variations have been previously reported. c.1844 T > C, another synonymous variation located in the *EXT1* exon, is linked with the pathogenic mutation c.1056+1G > A. Whether these variations interact remains to be further investigated.

## Data Availability

The data used to support the findings of this study are included within the article. If additional details are required, please contact the corresponding author.
